# Genetic analysis of the blood transcriptome of young healthy pigs to improve disease resilience

**DOI:** 10.1186/s12711-023-00860-9

**Published:** 2023-12-12

**Authors:** Kyu-Sang Lim, Jian Cheng, Christopher Tuggle, Michael Dyck, PigGen Canada, Frederic Fortin, John Harding, Graham Plastow, Jack Dekkers

**Affiliations:** 1 Department of Animal Science, Iowa State University, Ames, IA USA; 2 Department of Agricultural, Food and Nutritional Science, University of Alberta, Edmonton, AB Canada; 3 PigGen Canada Research Consortium, Guelph, ON Canada; 4 Centre de Développement du Porc du Québec Inc. (CDPQ), Québec City, QC Canada; 5 Department of Large Animal Clinical Sciences, University of Saskatchewan, Saskatoon, SK Canada; 6 Department of Animal Resource Science, Kongju National University, Yesan, Chungnam Republic of Korea

## Abstract

**Background:**

Disease resilience is the ability of an animal to maintain productive performance under disease conditions and is an important selection target. In pig breeding programs, disease resilience must be evaluated on selection candidates without exposing them to disease. To identify potential genetic indicators for disease resilience that can be measured on selection candidates, we focused on the blood transcriptome of 1594 young healthy pigs with subsequent records on disease resilience. Transcriptome data were obtained by 3’mRNA sequencing and genotype data were from a 650 K genotyping array.

**Results:**

Heritabilities of the expression of 16,545 genes were estimated, of which 5665 genes showed significant estimates of heritability (*p* < 0.05), ranging from 0.05 to 0.90, with or without accounting for white blood cell composition. Genes with heritable expression levels were spread across chromosomes, but were enriched in the swine leukocyte antigen region (average estimate > 0.2). The correlation of heritability estimates with the corresponding estimates obtained for genes expressed in human blood was weak but a sizable number of genes with heritable expression levels overlapped. Genes with heritable expression levels were significantly enriched for biological processes such as cell activation, immune system process, stress response, and leukocyte activation, and were involved in various disease annotations such as RNA virus infection, including SARS-Cov2, as well as liver disease, and inflammation. To estimate genetic correlations with disease resilience, 3205 genotyped pigs, including the 1594 pigs with transcriptome data, were evaluated for disease resilience following their exposure to a natural polymicrobial disease challenge. Significant genetic correlations (*p* < 0.05) were observed with all resilience phenotypes, although few exceeded expected false discovery rates. Enrichment analysis of genes ranked by estimates of genetic correlations with resilience phenotypes revealed significance for biological processes such as regulation of cytokines, including interleukins and interferons, and chaperone mediated protein folding.

**Conclusions:**

These results suggest that expression levels in the blood of young healthy pigs for genes in biological pathways related to immunity and endoplasmic reticulum stress have potential to be used as genetic indicator traits to select for disease resilience.

**Supplementary Information:**

The online version contains supplementary material available at 10.1186/s12711-023-00860-9.

## Background

Disease resilience broadly refers to the ability of an individual to maintain intrinsic and biological functions against the external pathogenic environment. It is widely accepted that there is a large range of individual variation for disease resilience, supporting its value as an important trait to improve productivity in farm animals. However, it is difficult to predict the intrinsic disease resilience of individuals without exposure to disease, which is the challenge for improving disease resilience using nucleus breeding herds that require a high level of biosecurity. Biomarkers of resilience that can be obtained from blood collected at the nucleus herd level can help overcome this limitation.

Gene expression in blood has been quantified to understand the dynamic aspects of an organism, such as health status, response to infection, and biological processes related to various phenotypes of interest. Several previous studies in humans have revealed a sizable number of genes with heritable levels of expression in blood [1–3], and the relationships of heritability of gene expression with physical properties of the gene, such as their genomic location and gene length, have been reported [1, 3]. Moreover, mean heritability was found to be higher for genes related to immune system pathways [3]. In pigs, expression quantitative trait loci (eQTL) for genes expressed in the blood of healthy young pigs were identified using an expression genome-wide association study (eGWAS) [4], while immune response parameters in blood measured after vaccination against *Mycoplasma hyopneumoniae* showed moderate to high heritabilities [5]. These findings suggest that the immune capacity, as characterized by transcriptomes and immune traits in the blood of healthy young individuals, can be under genetic control. However, genetic prediction of disease resilience of individuals based on such measures has not been investigated in either humans or pigs.

We have previously reported on the use of a natural polymicrobial disease challenge study in pigs to investigate the use of natural antibody levels [6], the transcriptome [7], and the proteome [8] in the blood of visually healthy weaner pigs as indicators for resilience to disease. Interestingly, the blood transcriptome study, which was at the phenotypic level, showed that visually healthy nursery pigs with higher levels of expression of immune and stress response-related genes were less resilient after exposure to the polymicrobial pathogens in this natural disease challenge model [7]. Based on this evidence, we hypothesized that gene expression in blood could be a potential genetic indicator to predict disease resilience in pigs. We focused on gene expression in blood collected from young pigs after weaning and investigated not only the heritability of gene expression but also genetic correlations with disease resilience, which is represented by performance and clinical phenotypes measured on the pigs under the polymicrobial disease challenge. We used individual-level data for genotypes, gene expression, and resilience phenotypes for genetic analysis of transcriptome data and investigated the genetic architecture of gene expression-related disease resilience in pigs.

## Methods

### Study design

In total, 3205 pigs in 50 batches from the Natural Disease Challenge Model (NDCM) were used in the current study [9, 10]. Population-scale blood transcriptome data were generated from 1594 pigs in 37 batches. Briefly, in the NDCM, a batch consisting of 60 or 75 healthy weaned barrows (Yorkshire x Landrace) was moved to an experimental facility after weaning (~ 21 days of age) and acclimated for three weeks in a biosecure quarantine nursery (qNur) with a high level of biosecurity. Then, they were exposed to a natural polymicrobial disease challenge at a nearby challenge nursery (cNur) (3 to 4 weeks) and finisher (Fin) (up to slaughter), which were designed to mimic a commercial farm with high disease pressure. Pigs received no vaccinations, except for a PCV2 vaccine (Circoflex, Boehringer Ingelheim, St. Joseph MO), which was administered at entry into qNur. Strategic medications were used for about half of the batches to balance disease challenge levels with animal welfare.

### Performance and disease resilience phenotypes

To evaluate disease resilience, performance and clinical phenotypes, including subjective health scores, the number of individual therapeutic treatments, mortality, growth rate, feed efficiency, and carcass traits, were collected in the qNur, cNur, and Fin phases, and across the cNUR and FIN (All), on all 3205 pigs, including the 1594 pigs with gene expression data, as previously described [7, 9, 10]. Abbreviations of traits are represented by the combinations of information on phase (qNur, cNur, Fin, and All) and trait names.

Health scores (HS) were recorded on a 1 to 5 scale for each pig at four-time-points (qNurHS1, qNurHS2, NurHS, and FinHS), and converted into binary variables (0/1; 1 = pigs in perfect health; 0 = others) for the variance component analyses. For the treatment rate traits (TRT; NurTRT, FinTRT, and AllTRT), the numbers of individual therapeutic treatments were adjusted for the number of days during which a pig was present in each respective phase. Mortality (MOR) was recorded as 0 for pigs that survived and 1 for pigs that died during each challenge period (cNurMOR, cFinMOR, and AllMOR). The binary trait MT was defined by MOR combined with TRT (pigs that died versus pigs that survived without treatment) for each phase (cNurMT, FinMT, and AllMT) and was coded as 0 for pigs that survived and had no individual therapeutic treatment, 1 for pigs that died, and as missing for all other pigs. Average daily gain (ADG) was computed for each phase (qNurADG, cNurADG, and FinADG) to evaluate growth rate. Regarding feed efficiency measurements, average daily feed intake (ADFI), average daily feeding duration (ADFD), feed conversion ratio (FCR), and residual feed intake (RFI) were recorded in the finisher. ADFD is the average feeding time (duration) of daily records during the finishing period. RFI was computed by adjusting ADFI for average body weight, ADG in the finisher, and ultrasound backfat thickness, as described by [11]. LYLD was % lean meat, which was estimated using Destron backfat and muscle depth measurements taken at the slaughter plant, using the prediction equation derived in [12]. The carcass traits, i.e. carcass weight (CWT), dressing proportion (DRS), lean yield (LYLD), carcass backfat (CBF), and carcass loin depth (CLD) were recorded at a commercial slaughter facility.

All animals were genotyped with the 650 k Affymetrix Axiom Porcine Genotyping Array by Delta Genomics (Edmonton AB, Canada). Raw Affymetrix single nucleotide polymorphism (SNP) data were processed by Delta Genomics, separately for each group of the seven consecutive batches, with the Axiom Analysis Suite, using all default settings. The 435,172 SNPs and genotypes that passed quality control (minor allele frequency > 0.05, SNP call rate > 0.90, and individual call rate > 0.90) for all seven groups of the seven consecutive batches, on 3139 pigs, were used for analysis, as described by Cheng et al. [10].

### Blood RNA extraction and white blood cell count measurement

Blood samples were collected in the qNUR at ~ 27 days of age, using Tempus Blood RNA tubes (Thermo Fisher Scientific, USA) and then stored at -80 ℃ until RNA extraction. The RNAs were isolated using the Preserved Blood RNA purification kit I (Norgen, Canada) according to the manufacturer’s instructions. The RNA integrity number (RIN) of each extracted RNA was assessed by the 2100 Bioanalyzer (Agilent Technologies, USA) using the Eukaryote total RNA 6000 Nano kit. White blood cell (WBC) differentials were quantified on whole blood samples in K2 ethylenediaminetetraacetic acid (EDTA) tubes (Thermo Fisher Scientific, USA) taken at the same time, using the flow cytometry-based hematology analyzer (ADVIA®2120i Hematology System, Siemens Healthineers, Germany) according to the manufacturer’s instructions [13].

### 3’ mRNA sequencing with globin blocking

RNA-seq libraries were generated from ~ 500 ng of total RNA, using the QuantSeq 3' mRNA-Seq Library Prep kit FWD for Illumina with the RNA Removal Solution-Globin Block, *Sus scrofa*, according to the manufacturer’s protocol (Lexogen, Austria), as described by [7]. The constructed QuantSeq libraries were multiplexed using mRNA from up to 96 samples and sequenced in two batches. The first batch of samples was sequenced on the Illumina HiSeq 3000 Sequencing system (Illumina, USA), using single-end 50 bp sequencing to increase the sequencing depth [7]. The second batch was sequenced on the Illumina NovaSeq 6000 Sequencing system (Illumina, USA), using single-end 100 bp sequencing. Each library was sequenced on two lanes and the sequence reads obtained from the two lanes were combined.

### RNA-seq read processing and gene expression normalization

The raw QuantSeq reads were first processed using the BBDuk software (https://jgi.doe.gov/data-and-tools/bbtools/bb-tools-user-guide/bbduk-guide/) to trim the adapter sequences, poly-A tails, and low-quality bases, and to filter out reads shorter than 20 bp after trimming. Read quality before and after trimming was assessed using the FASTQC 0.11.5 software [14]. Then, trimmed reads were mapped to the *Sus scrofa* reference genome sequence (SSC11.1; Ensembl, http://www.ensembl.org/) using the STAR 2.5.3a software [15]. To overcome the high sensitivity of 3’mRNA sequencing to 3’end gene annotation, the optimized method described by [7] was used.

Reads that mapped to the globin genes *HBA* and *HBB* and to genes that had a zero-count in more than 80% of the samples were filtered out. The remaining read counts were normalized across the 1594 samples by the trimmed mean of M values (TMM) using the EdgeR package in R [16]. Then, a log2 transformation was applied to the normalized counts plus 1 to obtain scaled expression values for further analyses.

### Variance component analyses of gene expression

Variance components were estimated by restricted maximum likelihood (REML) using ASReml 4.0 [17]. The genomic relationship matrix, $\mathbf{G}$, was created as described by [10] by combining relationship matrices that were created separately for each of the seven companies based on method 1 of VanRaden [18], using the preGSf90 software [19], with relationships between companies set to zero. This allowed estimation of pooled within-company variance components, which are more relevant for closed breeding programs. The following general models with (WI) or without (WO) accounting for WBC composition were used in single-trait analyses to estimate variance components:

$${y}_{ijklm}={Batch}_{i}+{Enrich}_{j}+{Pen}_{k}+{Litter}_{ijkl}+{Age}_{ijklm}+{RIN}_{ijklm}+\left({COMP}_{ijklm}\right)+{u}_{ijklm}+{e}_{ijklm},$$

where ${y}_{ijklm}$ is the expression level; ${Batch}_{i}$ is a fixed batch effect ($i$ = 1, …, 50); ${Enrich}_{j}$ is the effect of provision of non-edible enrichment toys to some pens in qNur as part of another research project; ${Pen}_{k}$ is the random effect of pen by batch in qNur, with the vector $\mathbf{P}\mathbf{e}\mathbf{n}\sim N(\mathbf{0},\mathbf{I}{\sigma }_{P}^{2})$, where ${\upsigma }_{P}^{2}$ is the pen variance; ${Litter}_{ijkl}$ is the random litter environmental effect, with the vector $\mathbf{Litter}\sim N(\mathbf{0},\mathbf{I}{\sigma }_{L}^{2})$, where ${\upsigma }_{L}^{2}$ is the litter variance; ${Age}_{ijklm}$ is the covariate of age when the pig entered qNUR; ${RIN}_{ijklm}$ is the covariate of RIN;${COMP}_{ijklm}$ represents the covariates of the log2 of the proportion of each of the six WBC types (lymphocytes, neutrophils, monocytes, basophils, eosinophils, and large unstained cells), which were fitted only in the WI model to adjust gene expression by cell composition; ${u}_{ijklm}$ is the random additive genetic effect, with the vector $\mathbf{u}\sim N(\mathbf{0},\mathbf{G}{\sigma }_{A}^{2} )$, where $\mathbf{G}$ is the genomic relationship matrix and ${\upsigma }_{A}^{2}$ is the additive genetic variance; and ${e}_{ijklm}$ is the residual effect, with the vector $\mathbf{e}\sim N(\mathbf{0},\mathbf{I}{\sigma }_{e}^{2})$, where ${\upsigma }_{e}^{2}$ is the residual variance.

Correlation analyses of estimates of heritability and litter effects obtained from the WO and WI models were conducted using the R software. To identify genomic regions with more highly heritable gene expression levels, average heritability estimates of genes in non-overlapping windows of 0.5 Mb were investigated. Estimates of heritability and common environmental effects of gene expression in human blood [1] were used in a comparative analysis between pigs and humans.

### Genetic correlations of gene expression with performance and clinical disease traits

Gene expression data on genes with nominally significant (*p* < 0.05) heritability estimates based on models with or without cell composition were used to estimate genetic correlations with the 26 performance and clinical disease traits, with the latter collected on all 3205 animals. The survivor dataset described in [10] was used for these purposes, i.e. including data on pigs that survived until slaughter for traits other than mortality. Genetic correlations between gene expression and resilience phenotypes were estimated using bivariate models in ASReml 4.0 [17], with the model described above for gene expression and models described in [10] for the performance and disease resilience data.

### Functional enrichment analyses

The genes with heritability estimates higher than 0.02 for expression level were functionally annotated with gene ontology (GO) biological processes (BP), pathways, and disease annotation terms using the Multi Ontology Enrichment Tool (MOET) provided by the Pig Portal within the Rat Genome Database [20]. Gene set enrichment analyses (GSEA) for genetic correlation estimates were conducted using the GSEA_4.0.3 software [21], with gene sets based on GO biological processes (c5.bp.v7.1.symbols.gmt). For this purpose, gene symbols were converted from pigs to humans based on orthology information, and BP with 10 or fewer genes or with 500 or more genes in the dataset were filtered out.

The GSEA analyses were conducted separately for each analyzed performance and resilience phenotype using a gene list that was ranked by the genetic correlation estimates from the bivariate analyses, with the following standard options: number of permutations = 1000; collapse/remap to gene symbol = no_collapse; enrichment statistics = weighted. In total, 170 BP terms were significantly enriched for at least one trait (FDR < 0.05), and summarized by removing redundant GO terms based on the REVIGO tools [22]. Although many of these 170 BP were significant for only one of the 26 evaluated traits, their level of significance with all 26 traits was used to cluster them using the pheatmap package in R [23]. This clustering was based on the signed significance level (-log10(FDR)) of the enrichment of each of the GO terms with each evaluated trait, where the sign was based on whether an increase in expression of core genes in the GO term was associated with a favorable ( +) or unfavorable (−) change in the trait based on the estimates of the genetic correlations. Adding the sign to the significance level allowed BP terms to be clustered based on the direction of the genetic correlation of gene expression with the evaluated traits.

## Results

### Heritability and litter effects for gene expression levels in blood of young healthy pigs

**Fig. 1:**
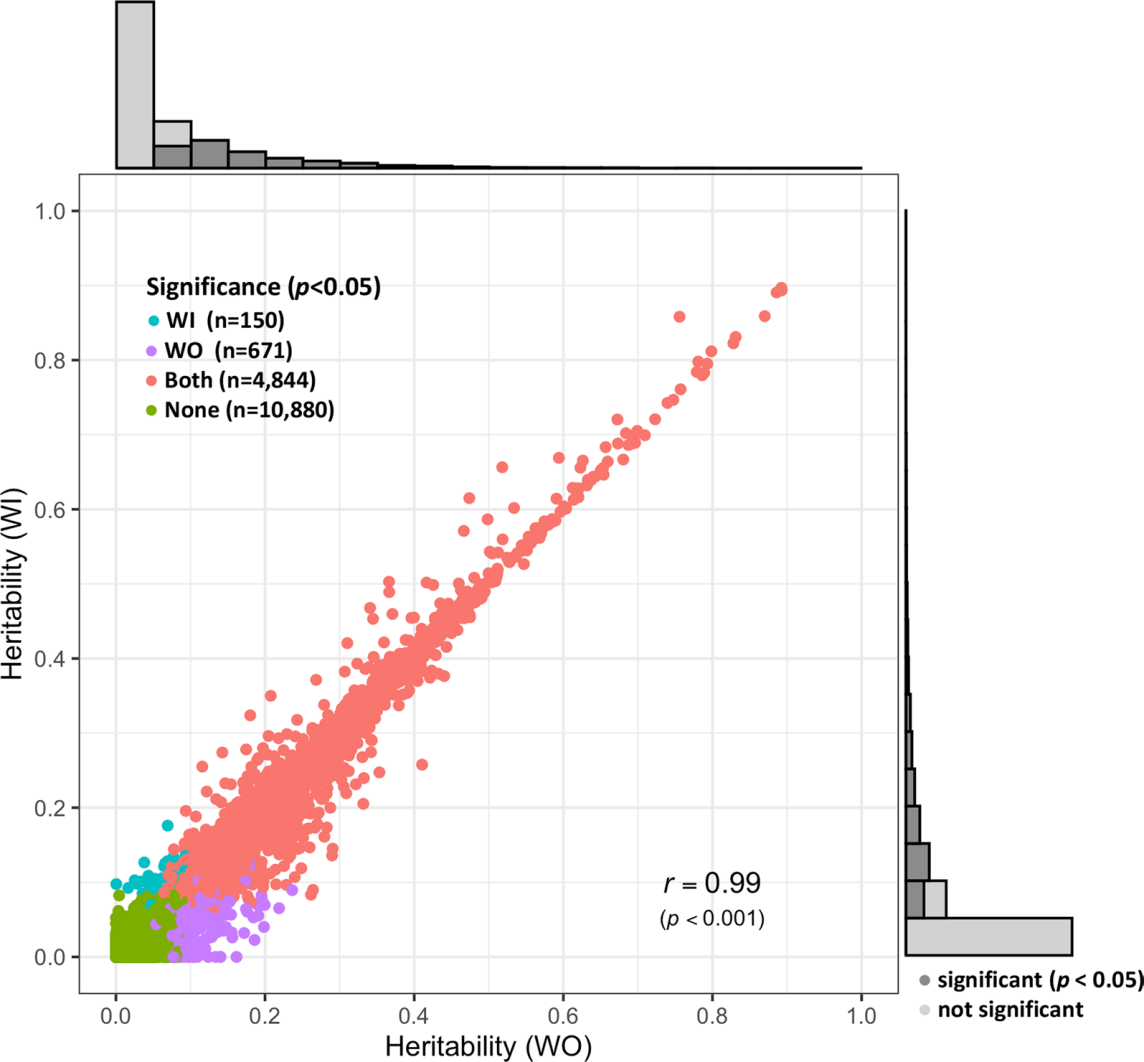
Distribution, significance, and comparison of estimates of heritability of expression levels for 16,545 genes based on models with (WI) or without (WO) adjustment for cell composition. Estimates were highly correlated between the two models. The dark gray bars in the histograms indicate the significant genes

Heritabilities of the expression level of 16,545 genes were estimated using the WI and WO models. Figure 1 shows the distributions of heritability estimates for the WI and WO models, which were both skewed to the right. Mean heritability was slightly lower for the WI than for the WO model (0.068 ± 0.001 versus 0.072 ± 0.001), as was the number of significantly heritable genes (*p* < 0.05) (4994 and 5515 for the WI and WO models, respectively, corresponding to 30 and 33% of all genes). Heritability estimates from the two models were highly correlated (*r* = 0.99). The top three heritable genes were *GPNMB*, *FAM178B*, and *MYL4*, with average heritability estimates based on the WI and WO models of 0.90, 0.89, and 0.89, respectively.

**Fig. 2:**
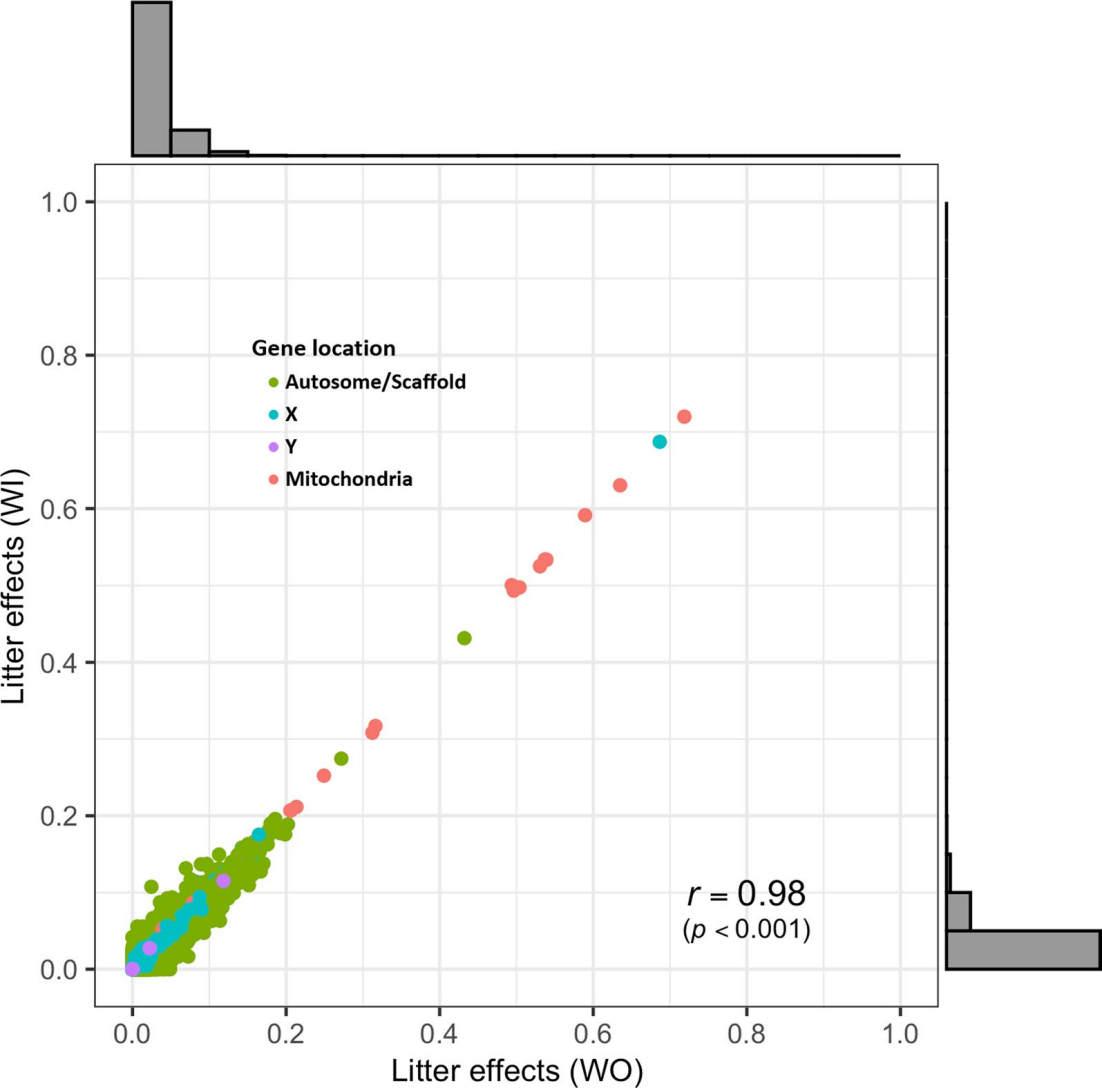
Distribution, significance, and comparison of estimates of the proportion of variance in expression levels for 16,545 genes explained by litter effects in models with (WI) or without (WO) adjustment for cell composition. None of the estimates were significant but they were highly correlated between the two models

The proportions of variance in gene expression that was explained by litter effects (*c*^2^) were estimated to be quite small, as indicated by the distribution of the estimates in Fig. 2. Average estimates were 0.022 ± 0.0002 and 0.023 ± 0.0003 for the WI and WO model, respectively, but estimates from the two models were highly correlated (*r* = 0.98). There were only 19 genes with an expression that was moderately affected by litter (*c*^2^ ≥ 0.2) for at least one model and most of these are mitochondrial genes (*ND1*, *ND2*, *ND5*, *COX2*, *ATP8*, and 10 tRNA genes), along with genes on *Sus scrofa* (SSC) chromosome 5 (XLOC_016362), 12 (*CCL5*), 15 (ENSSSCG00000034554), and X (ENSSSCG00000035520).

**Fig. 3:**
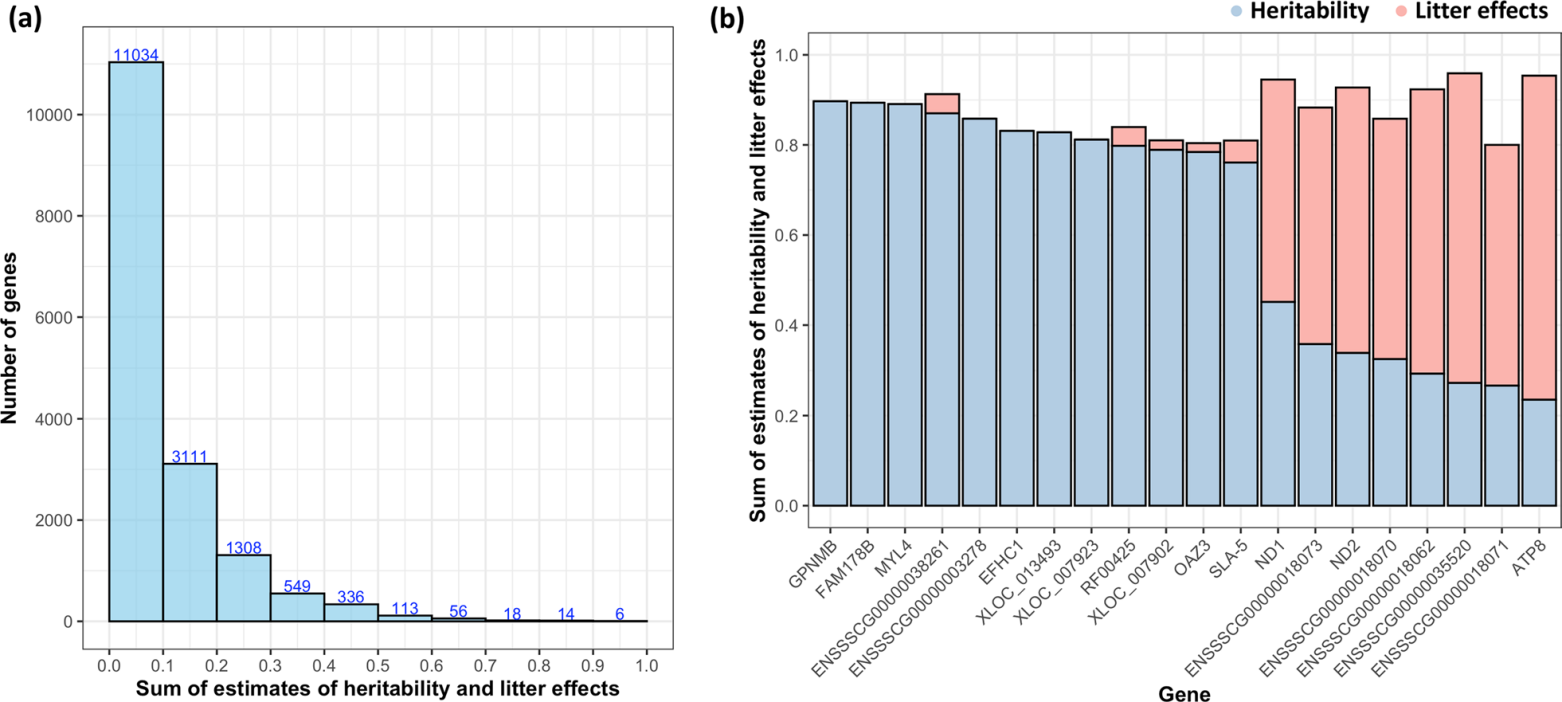
Distribution (**a**) and the top 20 genes (**b**) based on the sum of estimates of heritability and litter effects for the level of expression of 16,545 genes. The sum of estimates represents the highest values among the models with or without adjustment for cell composition

Considering the sum of the estimates of heritability and litter effects, the expression of some genes had very low residual environmental variance estimates (Fig. 3a), primarily because of high heritability estimates, with the top 20 genes shown in Fig. 3b. Interestingly, eight genes (7 on the mitochondrial genome and 1 on SSCX) had large litter effects and also sizeable heritability estimates.

### Genome characterization of transcriptome heritability

To identify trends depending on the physical properties of the genes, we investigated the relationship of the estimates of heritability of gene expression with gene position, average expression level, and gene length. The estimates of heritability of the level of gene expression showed weak but significant correlations with gene length (*r* = 0.10, *p* < 0.001) and average expression levels (*r* = 0.24, *p* < 0.001) (See Additional file 1: Fig. S1).

**Fig. 4:**
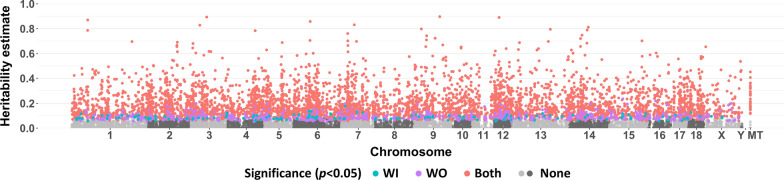
Manhattan plot of estimates of heritability of the level of expression of 16,545 genes by location across the genome and the mitochondrial (MT) genome and their level of significance. For each gene, the highest estimate from models with (WI) and without (WO) adjustment for cell composition was plotted. Genes located on scaffolds were excluded

**Fig. 5:**
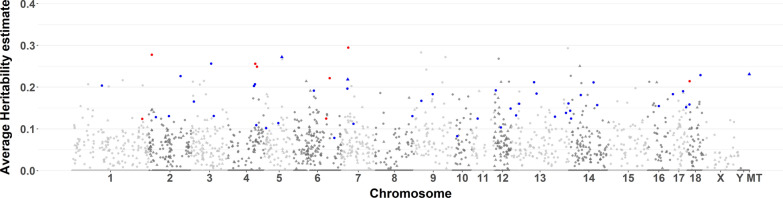
Manhattan plot of average estimates of heritability of the level of expression of genes in non-overlapping windows of 0.5 Mb across the genome and on the mitochondrial (MT) genome. Genes located on scaffolds were excluded. Each dot and triangle indicate a window that contained at least 5 or 10 expressed genes, respectively. Red and blue colors indicate windows for which, respectively, all or at least 80% of genes had expression levels that were significantly heritable (*p* < 0.05)

Figure 4 shows that genes with heritable expression levels were located across all SSC chromosomes and the mitochondrial genome. As expected, average heritability estimates for 0.5-Mb windows were more variable for windows with a small number of genes (See Additional file 2: Fig. S2). Therefore, only results for windows that contained at least five genes were plotted in Fig. 5. Windows for which at least 80% of genes had significantly heritable expression levels (*p* < 0.05) were considered heritable regions (54 windows). For six windows, all genes were significantly heritable, with an average heritability estimate higher than 0.2: at 11 Mb on SSC2; at 97 and 103 Mb on SSC4; at 128.5 Mb on SSC6; at 24.5 Mb on SSC7; and at 7 Mb on SSC18 (Fig. 5) (See Additional file 2: Fig. S2).

**Fig. 6:**
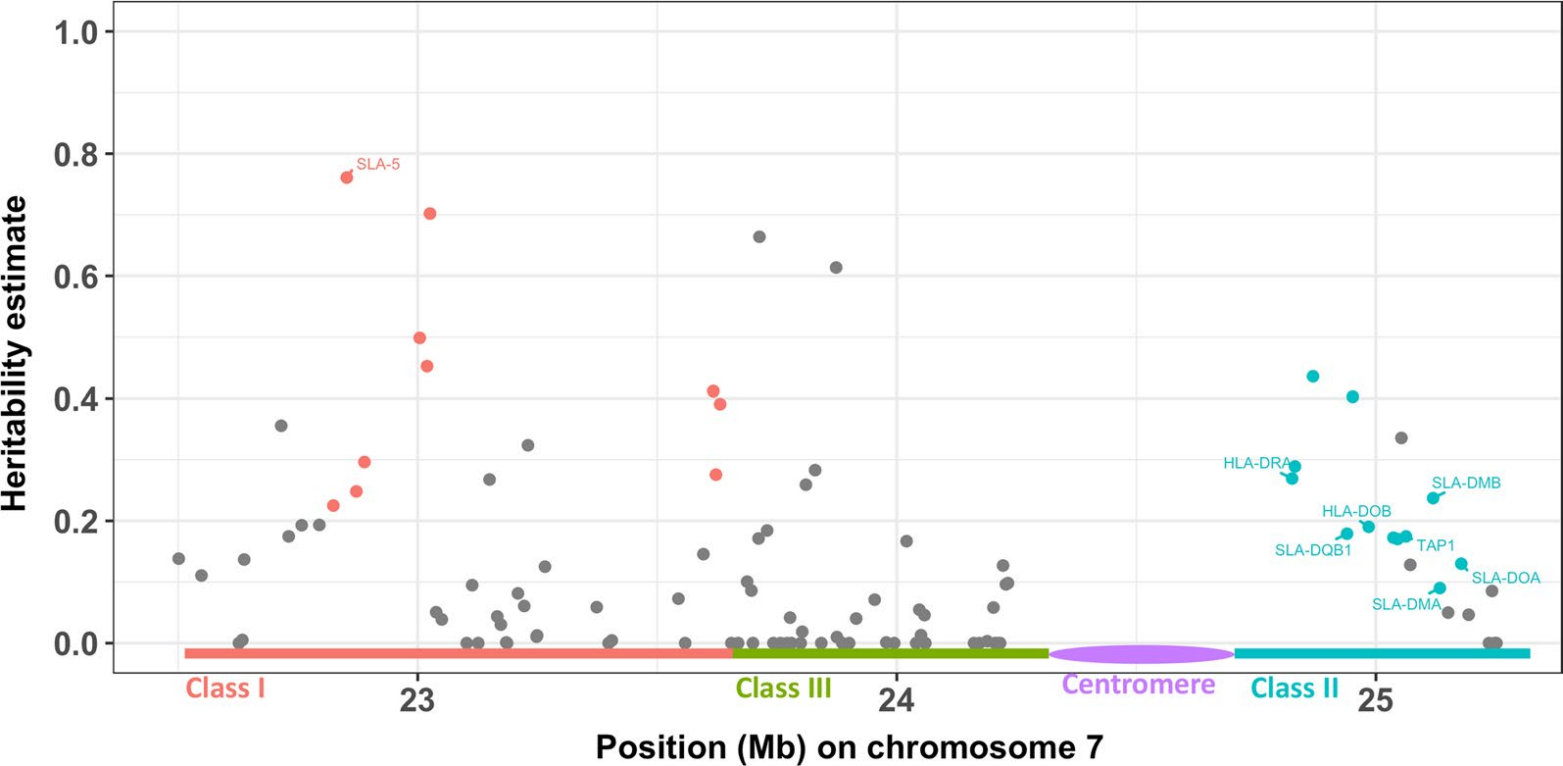
Manhattan plot of estimates of heritability of the expression of genes located in the swine leukocyte antigen complex region on chromosome 7. Red and blue dots indicate class I and II genes, respectively

Interestingly, one of these windows overlapped with the swine leukocyte antigen (SLA) complex (at 22.5 and 24.5 Mb on SSC7). Figure 6 shows estimates of heritability of the expression of genes located in the SLA complex. Note that not all genes in this region are SLA genes but the 10 class I SLA genes and the 12 class II SLA genes all had significantly heritable expression levels (*p* < 0.05). Among them, *SLA-5* showed the highest heritability estimate (0.76).

### Comparison with heritability of blood gene expression in human

**Fig. 7:**
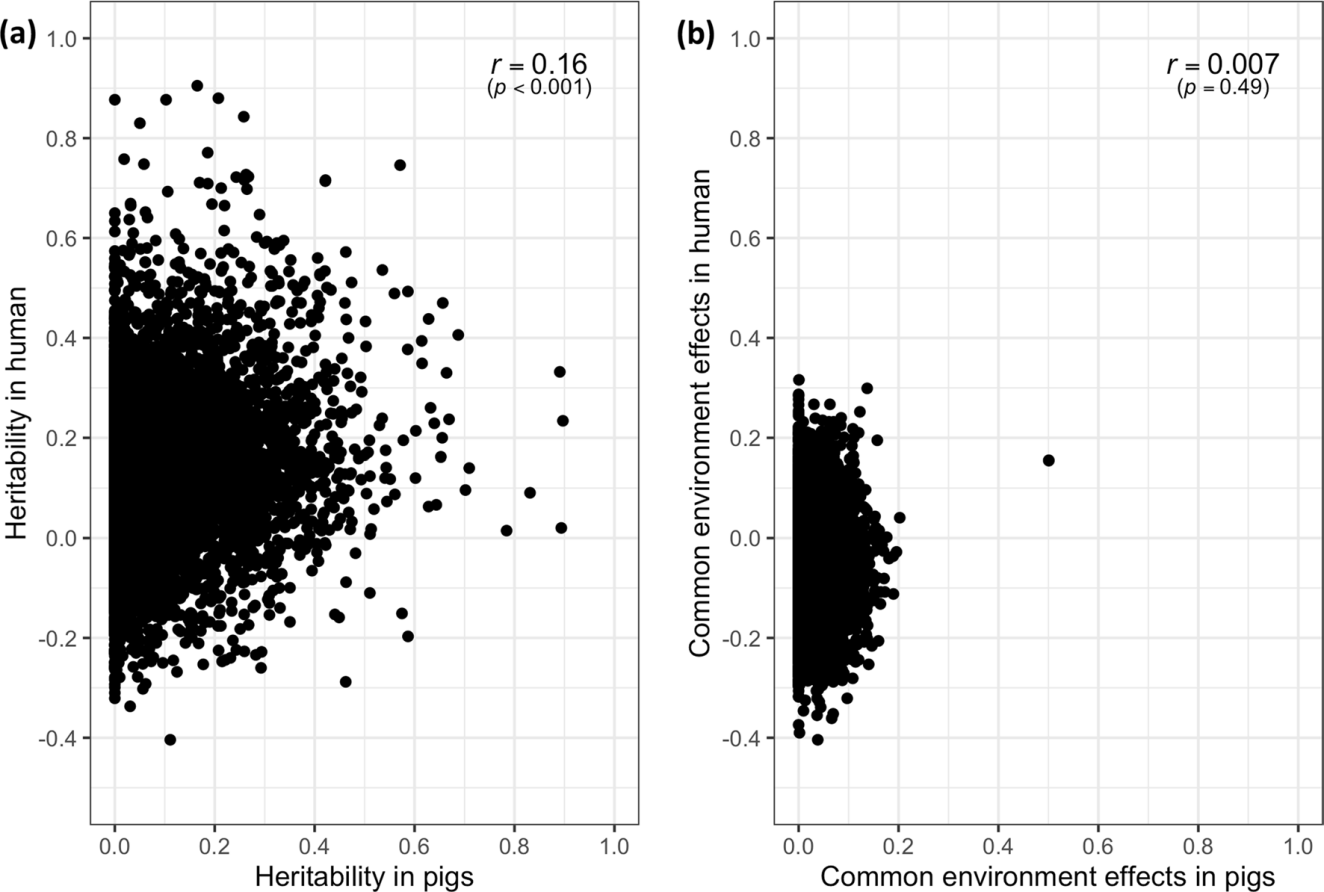
Comparison of estimates of heritability (**a**) and the variance explained by common environment effects (**b**) for the level of expression of comparable genes in the blood of pigs and humans

To compare heritabilities of gene expression levels between humans and pigs, we integrated human orthologous genes (n = 8605) and their twin-based estimates of heritability in the blood transcriptome of humans [1]. Figure 7 shows that the estimates of heritability of gene expression levels in humans and pigs were positively but weakly correlated (*r* = 0.16, *p* < 0.001), while the estimates of the proportion of variance explained by common environmental effects (i.e. litter effects in our results) were not significantly correlated (*r* = 0.007, *p* = 0.49). In spite of these weak relationships, some genes had an expression level that was highly heritable for both pigs and humans, including *CHI3L1* (0.75 in humans and 0.57 in pigs) and *CLU* (0.54 in both species). In addition, the expression of *ND1* had sizeable common environmental effects for both species (0.16 and 0.50 for humans and pigs, respectively).

### Functional annotation of genes with heritable levels of expression

**Fig. 8:**
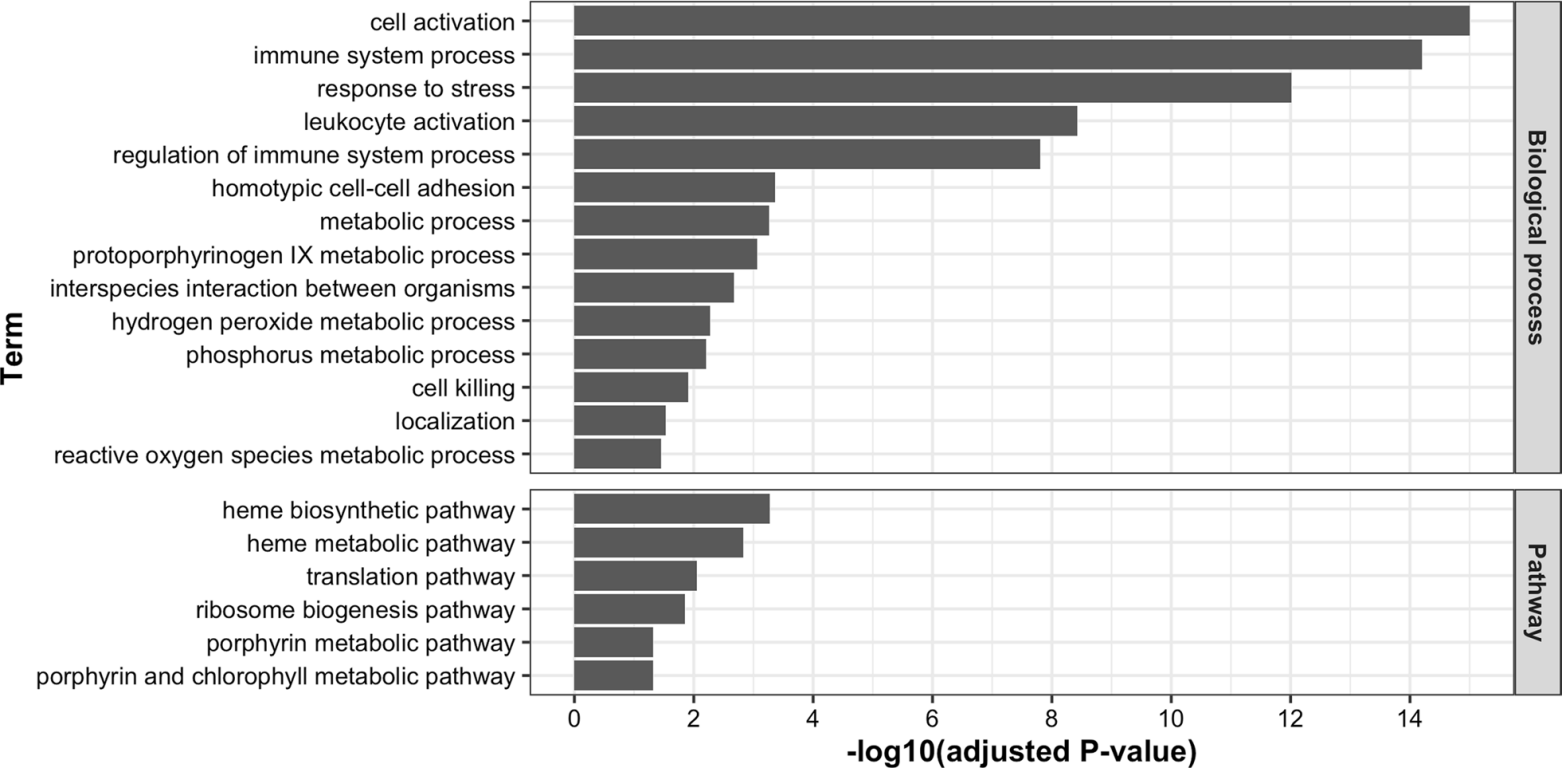
Functional enrichment analysis of genes with heritability estimates of expression higher than 0.2 for gene ontology biological processes and pathways based on the pig portal within the rat genome database. Significant biological process terms (FDR < 0.05; n = 104) were grouped based on semantic similarity by REVIGO, which yielded 14 over-represented terms

The level of expression of 1675 genes had heritability estimates of 0.2 or more (See Additional file 3: Fig. S3). The 1365 genes that had annotated functions among these were investigated for enrichment of GO terms, and we identified 104 significantly (FDR < 0.05) enriched GO biological processes, as illustrated in Fig. 8, with their representative terms based on semantic similarity, as determined by the REVIGO algorithm [20]. The biological processes of cell activation, immune system process, response to stress, leukocyte activation, and regulation of immune system process showed the most significant enrichments (FDR < 1.0 × 10^–7^). Metabolic processes such as protoporphyrinogen IX, hydrogen peroxide, phosphorus, and reactive oxygen species were also enriched among the more heritable genes. Figure 8 also shows pathways that were significantly enriched among the more heritable genes, which included the porphyrin and heme metabolism-related pathways and the translation-related pathway (See Additional file 3: Fig. S3).

**Fig. 9:**
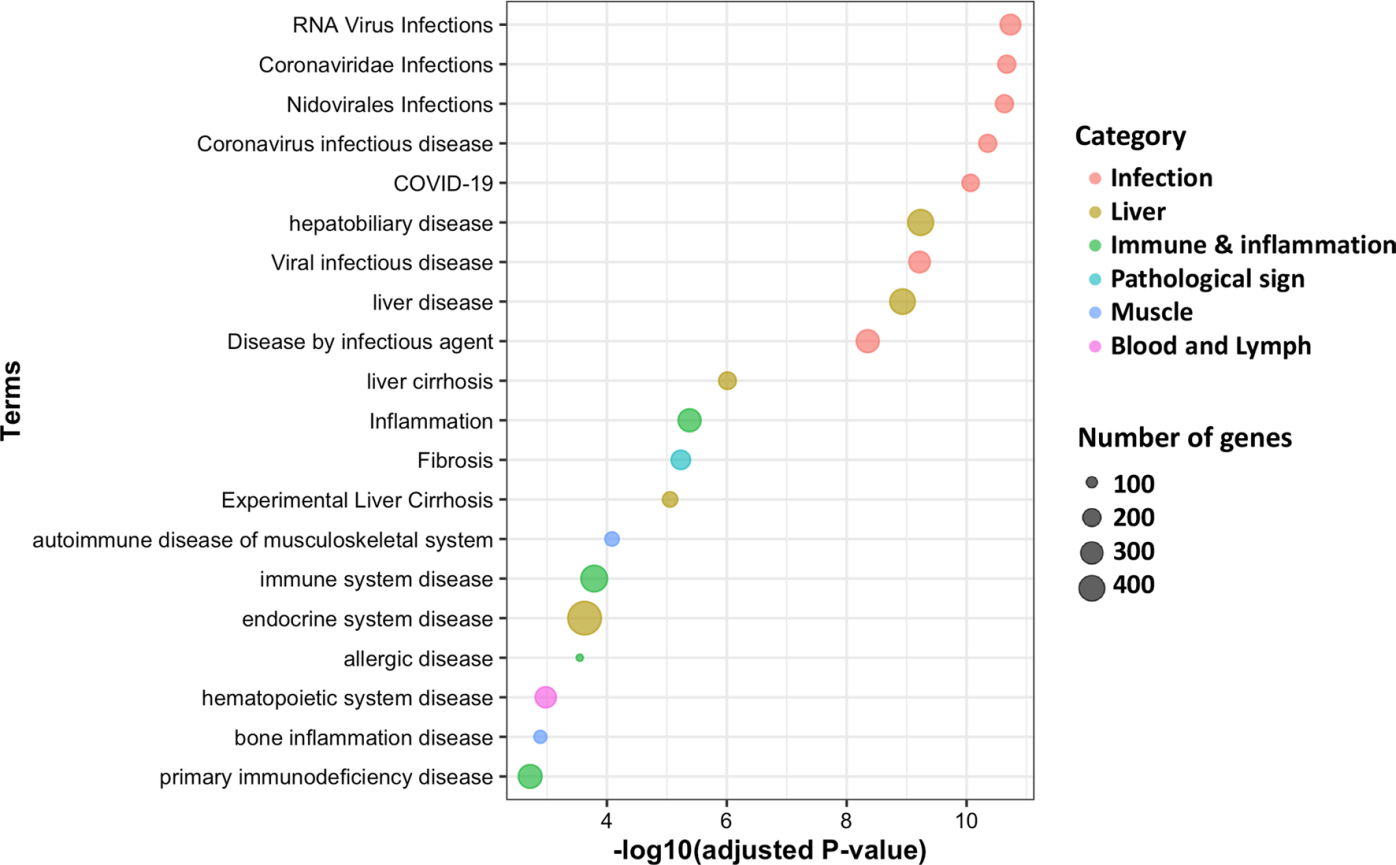
Top 20 disease annotation terms of the pig portal within the rat genome database that were significantly enriched for genes with heritability estimates of expression greater than 0.2

Figure 9 shows infectious disease-related terms that were most significantly enriched among the more heritable genes (FDR < 1.0 × 10^–8^). Interestingly, the more heritable genes were enriched for RNA virus infections, including SARS-CoV2, implying possible genetic differences in host immunity against COVID-19 disease. Similar to the enriched GO biological processes, immune and inflammation-related disease terms were significantly enriched among the more heritable genes (FDR < 0.01). Figure 9 also shows that the more heritable genes were associated with a wide range of diseases, including liver disease, pathological signs, musculoskeletal disease, hemic and lymphatic disease, and respiratory system disease.

### Genetic correlations of gene expression with performance and clinical disease traits

**Fig. 10:**
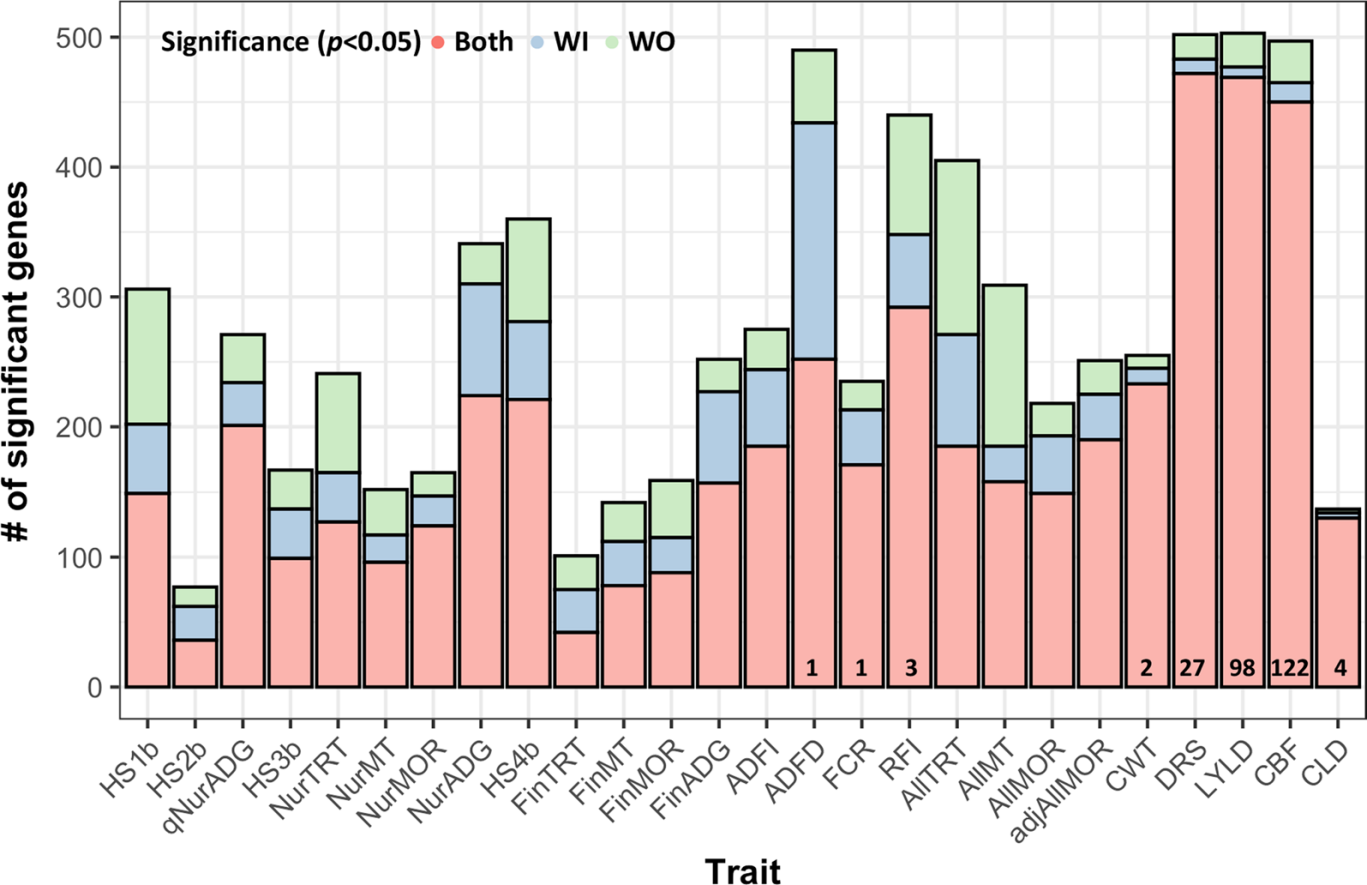
The number of genes with a level of expression that was significantly genetically correlated with performance and clinical disease traits for models with (WI) or without (WO) adjustment for cell composition. The numbers in the bars indicate the number of significant genes with an FDR < 0.20 based on the WO and/or WI models

In total, 5665 genes with nominally significant (*p* < 0.05) heritability estimates based on models with or without cell composition were investigated for further analysis of genetic correlations of their expression levels with 26 performance and clinical disease traits that were collected in qNur, cNur, and Fin. Figure 10 shows the numbers of genes with an expression level that had nominally significant (*p* < 0.05) genetic correlations with each performance and clinical disease phenotype. However, genes that showed significant genetic correlation estimates with an FDR < 0.20 were limited to the feed efficiency traits ADFD (n = 1), FCR (n = 1), and RFI (n = 3), and to the carcass traits carcass weight (CWT, n = 2), DRS (n = 27), LYLD (n = 98), CBF (n = 122), and CLD (n = 4). Genes with expression levels that had highly significant genetic correlation estimates (> 0.65) were XLOC_005262 with FCR, *PER3* and *BATF2* with RFI, XLOC_001578, ENSSSCG00000004415, XLOC_021659, and *GORASP2* with LYLD, and XLOC_001578, ENSSSCG00000004415, and XLOC_021659 with CBF.

### Gene set enrichment analysis of genetic correlations across genes and phenotypes

**Fig. 11:**
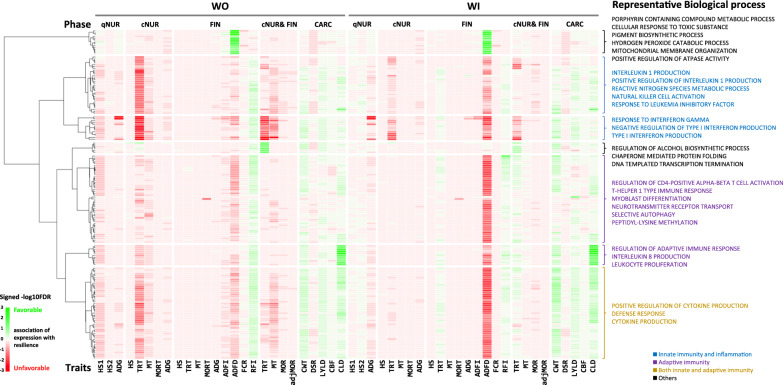
Biological processes (n = 170) that were significantly enriched (FDR < 0.05) among the genes ranked by p-values and directions of the genetic correlation estimates with at least one performance or clinical disease phenotype. Color intensity represents the significance (− log10(FDR)) of the enrichment and green/red color indicates that an increase in the expression of core genes in that biological process had favorable/unfavorable genetic correlations with the trait

Although there were sizable numbers of genes with significant genetic correlation estimates (FDR < 0.20) for carcass traits, the statistical power to estimate genetic correlations at the individual gene level was limited for most traits. Thus, to determine whether the level of expression of genes that are associated with certain biological processes exhibited directional genetic correlation estimates with certain performance and disease resilience traits, GSEA were conducted. For this purpose, for each recorded phenotype, the 5665 genes were ranked based on the signed *p*-value of their genetic correlation estimate with the trait, with the sign of the p-value representing that of the corresponding genetic correlation estimate, such that the genes were ranked from those that had the most significant positive to those with the most significant negative genetic correlation estimate. Figure 11 shows the GSEA results for 170 GO biological processes that were significantly (FDR < 0.05) enriched for at least one performance or clinical disease phenotype. Hierarchical clustering of the BP based on the signed significance (− log10(FDR)) of their enrichment, identified seven clusters, of which five were related to innate and/or adaptive immunity.

These enriched immune-related clusters generally showed an unfavorable relationship with HS, TRT, and ADFD, which indicates that the higher expression of genes annotated for BP in these clusters tended to show unfavorable genetic correlations with these phenotypes. In contrast, these clusters generally showed a favorable relationship with RFI, CWT, and CLD. The cluster at the bottom of the heatmap of Fig. 11 consists of cytokine production and defense response, which are involved in both innate and adaptive immunity and showed similar relationships with HS, TRT, ADFD, RFI, CWT, and CLD as described for the immune-related clusters. The clusters of innate immunity and inflammation showed stronger enrichment for TRT recorded in cNur than for the other traits, while more terms in the clusters of adaptive immunity appeared significant for ADFD, which was measured in the finisher. The innate immunity cluster that contains interferon-related terms showed significant enrichments for ADG in qNur as well. The term of positive regulation of neuron differentiation in the adaptive immunity cluster showed unfavorable genetic correlations with mortality in the finisher for both the WO and WI models. The adaptive immunity cluster with interleukin 8 production and leukocyte proliferation showed clear favorable genetic relationships with CLD.

Various non-immune functions were enriched for ADFD, including the porphyrin containing compound metabolic process, cellular response to toxic substance, the pigmentation biosynthetic process, the hydrogen peroxide catabolic process, mitochondrial membrane organization, and positive regulation of ATPase activity. Moreover, TRT across the challenge nursery and finisher was favorably related to regulation of alcohol biosynthetic process, chaperone mediated protein folding, and DNA templated transcription termination.

## Discussion

The objective of this study was to investigate the blood transcriptome of young healthy pigs as a source of potential genetic indicators for disease resilience. To be useful for genetic improvement, indicator traits must be heritable and have a sizeable genetic correlation with the target trait(s), i.e. disease resilience in this case. To our knowledge, this represents the largest quantitative genetic analysis of gene expression in livestock in terms of the number of animals with RNAseq data. It is also the first study to comprehensively investigate the use of blood transcriptome data on healthy animals as genetic indicators for disease resilience.

### Heritability and common environmental effects of gene expression

Expression of 16,545 genes was detected in blood samples of the analysed population of young and visually healthy pigs, of which 5665 genes showed significantly heritable expression levels (*p* < 0.05), supporting their potential usefulness as genetic indicators for selection in pigs. Estimates of heritability from the models with (WI) and without (WO) correction for cell composition were highly correlated (*r* = 0.99). The most significant heritability estimates were detected for the WO model (n = 5515) but the expression of 150 genes was significant for the WI model but not for the WO model (Fig. 1), suggesting the utility of the adjustment for the cell composition in the genetic analysis of blood transcriptome data, at least for these genes.

Genes such as *GPNMB*, *FAM178b*, and *MYL4* showed the highest heritability estimates (around 0.9) (Fig. 3b). The expression of these genes also showed genetic correlations with resilience phenotypes such as CLD and ADFD. Previous studies in humans have identified relationships of these three genes with familial diseases such as Alzheimer’s disease [24], bipolar disorder [25], and atrial cardiomyopathy [26], respectively. Therefore, the high heritability of the level of expression of these genes in early life and their genetic correlations with disease resilience phenotypes suggest that genetic factors could play a role in determining the risk of pigs developing these or related health problems.

Compared to estimates of heritability, the proportions of variance in gene expression that were explained by common environmental (litter) effects were limited, except for several mitochondrial genes and several genes on the sex chromosomes (Fig. 2). The limited litter effects on autosomal genes reflects the nature of gene expression at a given stage (~ 1 week after weaning and ~ 40 days of age) when passive immunity transmitted from the sow decreases and active immunity of the piglet itself increases. This is supported by the higher heritability estimates and smaller litter effects for levels of IgM compared to IgG natural antibodies in blood taken at the same time on these pigs, as described by [6]. The IgG but not the IgM isotype can be transmitted from the mother to the fetus via the placenta in humans [27] and through the colostrum in cattle [28] and pigs [29]. However, one autosomal gene, i.e. *chemokine (C–C motif) ligand 5* (*CCL5*) had an expression level that was sizably affected by litter effects (estimates of 0.20 and 0.19 based on the WO and WI models, respectively). Previous reports have shown that maternal immune activation during pregnancy can increase the mRNA expression level of *Ccl5* in the embryonic brain in mice [30, 31], supporting a significant litter effect for *CCL5* observed here in young pigs. The expression of *CCL5* was also heritable, with estimates of 0.17 and 0.21 based on the WO and WI models, respectively, and had significant favorable genetic correlation estimates with LYLD and CBF.

The larger common litter effects that were observed for multiple genes on the mitochondrial genome and sex chromosomes reflect that mitochondrial DNA and the single copies of the X and Y chromosomes that males carry originated from a single parent, while the model of analysis assumes bi-parental inheritance of gene expression levels. Thus, at least part of the observed litter effects for these genes may reflect genetic effects of (cis-)eQTL on the mitochondrial genome or sex chromosomes that were not picked up by the bi-parental genetic effect assumed in the analysis model. Interestingly, the expression of mitochondrial genes such as *ND1*, *ND2*, and *ATP8* also had sizeable estimates of heritability (Fig. 3b). This implies that at least some of the eQTL for mitochondrial genes are autosomal, which is supported by previous eQTL studies for mitochondrial genes in humans [32] and pigs [33]. Note that some of the litter effects that were observed for autosomal genes could also be caused by eQTL that are located on mitochondrial DNA and sex chromosomes.

### Genomic and functional characterization of transcriptome heritability

Studies on blood in humans have reported enrichment of genes with heritable levels of gene expression in gene-poor regions of the genome [1] and positive correlations of estimates of heritability for a gene with its length and its level of expression [3]. We also observed positive relationships of gene length and the level of gene expression with estimates of heritability (See Additional file 1: Fig. S1) but their magnitude was small in our data. In addition, in our results for pigs, genes with heritable levels of expression were spread across the chromosomes (Fig. 4) and the number of expressed genes in a 0.5-Mb window was not significantly associated with the average heritability of the levels of expression of genes in the window (See Additional file 2: Fig. S2). Therefore, we focused on 0.5-Mb windows with at least five expressed genes to identify heritable gene regions (Fig. 5). The window with the highest average heritability (0.29) was at 24.5–25 Mb on SSC7. This window contains the SLA class II genes (Figs. 5 and 6), i.e. *HLA-DRA*, *SLA-DRB1*, *SLA-DQA1*, *SLA-DQB1*, and *HLA-DOB,* which all had significant (*p* < 0.001) estimates of heritability. These genes show a high level of homology to human leukocyte antigen (HLA) class II genes [34]. In humans, the levels of expression of *HLA-DRA* and *HLA-DOB* in blood were also significantly heritable (*p* < 0.01) [1].

Heritability estimates of the level of expression in blood of orthologous genes between pigs and humans showed a weak positive correlation (Fig. 7), possibly because of species differences and differences in blood sampling times and conditions. Previous studies on humans were conducted on the blood from healthy adult individuals [1, 3], while we focused on the blood from healthy young or juvenile pigs. However, the positive relationship between estimates of heritability of orthologous genes and the sizable number of genes (n = 1125) that had significant heritability estimates for expression level (*p* < 0.05) for both pigs and humans implies some similarities in the genetics of gene expression in blood between pigs and humans.

Genes with heritable levels of gene expression (estimates higher than 0.2) were enriched for various immune-related biological processes based on GO terms such as cell activation, immune system process, response to stress, and leukocyte activation (Fig. 8). This likely reflects that the piglets were developing their own active immunity, in a natural process associated with aging and possibly in response to several common stressors that they experienced, including weaning, transportation, mixing, and new feed ingredients. Interestingly, the infectious disease-related annotations provided by the Rat Genome database showed the highest enrichment for genes with heritable levels of gene expression (Fig. 9). The heritable expression levels in the blood of young healthy pigs for genes related to immunity and disease infection implies that host genetics can contribute to variation in disease resilience in pigs. The heritable expression of disease-related genes in pigs can also have implications for the use of the pig as a biomedical model for humans [35, 36]. The highly significant enrichment of genes that were annotated for COVID-19 among the genes with heritable expression levels support previous studies on the role of host genetics on the susceptibility of humans to COVID-19 infections using GWAS and candidate gene approaches [37].

### Genetic correlations of the blood transcriptome with disease resilience

Using a subset of the data used here, Lim et al. [7] described phenotypic associations of the level of gene expression in the blood of young healthy pigs with the subsequent resilience of these pigs to disease. Here, we further investigated the genetic basis of these associations, by estimating genetic correlations of gene expression levels with performance and disease resilience traits, focusing on 5665 genes with heritable expression levels (*p* < 0.05).

Estimating the genetic correlation between gene expression levels and phenotypes, as in our study, is considered the “gold standard” for identifying genes with a level of expression that is genetically correlated with a target phenotype [38]. As a proxy, Gusev et al. [38] used so-called transcriptome-wide association studies (TWAS) to identify such genes. In TWAS, a training dataset with gene expression and whole-genome SNP genotype data is used to develop genomic predictions for the expression of each gene, which are then used to predict the level of expression of genes across the genome in a dataset consisting of individuals that have SNP genotypes and phenotypes for the target trait. The resulting genomic predictions of gene expression are then correlated with the target phenotype. Significant correlations are expected to be the result of genetic effects that affect both the level of gene expression and the target phenotype. However, they are not directly comparable to genetic correlations, which quantify correlations between true genetic values for pairs of traits. In the present study, we used a large dataset with SNP genotypes and target phenotypes, of which a subset also had gene expression data. For such a data structure, direct estimation of genetic correlations using phenotypes for multiple traits on relatives is well accepted to be the gold standard to quantify genetic correlations between traits [39].

Significant genetic correlations (*p* < 0.05) of the level of expression for some genes were observed for each performance and resilience phenotype investigated (Fig. 10) but the number of genes with low false discovery rates was limited for most traits. To overcome the limited power of the estimation of genetic correlations for individual genes, we used GSEA to combine evidence of directional genetic correlations of the level of expression of genes that are associated with certain biological processes (Fig. 11). In general, the directions of enrichments were similar for the GSEA results based on genetic correlations estimated using the WO and WI models for gene expression, but the magnitude of significance levels was lower for the WI model, except for genetic correlations with ADFD and CLD, implying that cell composition affects the transcriptome data in blood and can also impact the genetics of disease resilience. The level of expression of genes involved in biological processes related to innate and adaptive immunity showed unfavorable genetic correlations with resilience phenotypes measured in the challenge nursery and finisher, except for RFI (Fig. 11). In other words, under the circumstances inherent to this trial, visually healthy piglets with higher levels of expression of immunity-related genes in blood may be less resilient genetically when exposed to major pathogens. A similar relationship was identified at the phenotypic level by Lim et al. [7]. Corresponding to the general sequence of immune response, biological processes related to innate immunity showed strong enrichment for genetic correlations with resilience traits during the challenge nursery, especially for TRT, MT, and growth rate, while biological processes related to adaptive immunity were more significant for genetic correlations with feed intake related traits in the finisher, such as ADFI and ADFD. The cytokine-related terms across innate and adaptive immunity, including interleukins (1 and 8) and interferons (type I and type II), indicated the usefulness of their expression levels as genetic indicators of disease resilience. An inflammatory response is generally triggered to remove foreign substances or pathogens that invade from outside, but excessive inflammation can harm health, accelerate aging, and cause damage to organs [40]. Hence, the higher expression levels of innate immunity-related genes observed in the blood of some visually healthy piglets may be due to their susceptibility to common stressors after weaning and may induce excessive activation of inflammation response and clinic symptoms when they are exposed to pathogens.

Interestingly, our GSEA results showed an enrichment of genes associated with several interferon related biological processes among genes with negative genetic correlations with growth rate in the quarantine nursery, i.e. prior to the exposure of pigs to pathogens, which may reflect another biological mechanism that may be associated with genetics of disease resilience. Hodes et al. [41] reported that susceptibility to social stress was related to individual differences in cytokine profiles in blood, supporting a possible relationship between blood gene expression pattern in these young pigs following weaning and their future disease resilience. They [41] suggested that the P62 protein suppressed inflammation, but in our data the level of expression of its coding gene, *SQSTM1*, had a very low heritability estimate (< 0.03 from both models). Nevertheless, genes involved in the inhibition of excessive inflammation could be a good start point for further studies that target disease resilience.

The biological process of the positive regulator of neuron differentiation was enriched for favorable genetic correlations with mortality in the finisher (Fig. 11). Several common infectious diseases in swine are associated with central nervous disorders, such as Glässer's disease and Aujezsky's disease [42]. Glasserella paresis infection was confirmed to be present in the NDCM. This suggests that the expression level of genes associated with the neural system as a response to non-infectious stressors in young healthy pigs could be a target for evaluating their resilience to infectious disease in the future.

Interestingly, the higher expression of genes associated with the biological process of chaperone mediated protein folding was genetically associated with lower treatment rates across the challenge nursery and finisher periods (Fig. 11). The expression of *HSPH1*, one of the core genes in this enriched gene set, had significant genetic correlations with AllTRT (*r*_*g*_ = -0.62 ± 0.30 and -0.66 ± 0.29 for the WO and WI modes, respectively). Several previous studies have reported that the expression of *HSPH1* was altered during infection with porcine reproductive and respiratory syndrome virus [43, 44] and with porcine circovirus type 2 [45], supporting the genetic association of *HSPH1* expression in healthy young pigs with lower health treatment rates in the current study.

The expression of genes involved in the regulation of adaptive immune response showed enrichment among genes with favorable genetic correlations with CLD (Fig. 11). Among the core enriched genes for the regulation of adaptive immune response, three genes, i.e. *ANXA1*, *CD1D*, and *IL10*, showed significant genetic correlations with CLD for both the WO and WI models for gene expression. A favorable phenotypic association of the level of annexins A1 coded by the *ANXA1* gene with resistance to respiratory disease was reported in weaned beef calves by [46]. Annexin A1 has an anti-inflammatory activity by inducing macrophages to secrete the anti-inflammatory cytokine IL-10 [47]. These results suggest that a higher expression of *ANXA1* and *IL10* in the blood of young healthy pigs may be associated with a reduction of the deleterious effects of inflammatory responses when exposed to infectious disease, helping to maintain the ability to produce lean meat towards market age.

Our finding that certain GO terms were enriched among the genes with a level of expression that had larger (positive or negative) genetic correlations with performance and disease resilience traits, suggests that the blood transcriptome of young healthy pigs can be used to predict breeding values for these traits. Several methods to incorporate intermediate -omics traits in genomic prediction of breeding values for traits of interest have recently been developed [48, 49]. These methods are being applied to these data in ongoing studies.

## Conclusions

In a previous study, we identified phenotypic associations between the blood transcriptome of healthy young pigs and their subsequent disease resilience [7]. Here, a larger population-scale transcriptome and phenotype dataset was generated and we focused on the genetic analysis of the blood transcriptome and its genetic correlations with subsequent resilience phenotypes. We identified genes with heritable expression levels in blood of young and visually healthy pigs. These genes were spread across the genome but the SLA region was identified as one of the genomic regions where the heritable genes were clustered. The genes with a heritable expression were enriched for various disease-related terms. In addition, we detected genetic correlations of the blood transcriptome of visually healthy weaned piglets with disease resilience, represented by clinical and performance traits under a natural polymicrobial disease challenge. Gene set enrichment analyses suggested that the expression levels of genes related to innate and adaptive immunity in the blood of visually healthy weaned piglets are genetically correlated with disease resilience. Taken together, this study supports the possible use of the blood transcriptome of healthy weaned piglets as a genetic indicator to select for disease resilience in pigs, although further research is needed to confirm these associations and crystalize the salient features of the blood transcriptome of young healthy pigs.

### Supplementary Information

**Additional file 1: Figure S1.** Relationship of the estimates of heritability of gene expression with gene length (a) and average expression level (b).

**Additional file 2: Figure S2.** Box blots of average heritability estimates of genes in 0.5-Mb non-overlapping windows across the genome, versus the numbers of expressed genes within the window.

**Additional file 3: Figure S3.** Number of genes (n = 1675) with expression levels that had estimates of heritability of 0.2 or higher in the models with (WI) or without (WO) accounting for the cell composition.

## Data Availability

The data were generated on commercially owned animals and, therefore, contains proprietary information. As a result, the data analysed in this study are not publicly available but are stored in a secure data base at the University of Alberta and they can be made available by the corresponding author on reasonable request.
