# Supplementary figures and images for: Genetic analysis of the blood transcriptome of young healthy pigs to improve disease resilience
Source: Genet Sel Evol. 2023 Dec 12;55:90. doi: 10.1186/s12711-023-00860-9 (PMC10714454; doi:10.1186/s12711-023-00860-9)

## Slide 1
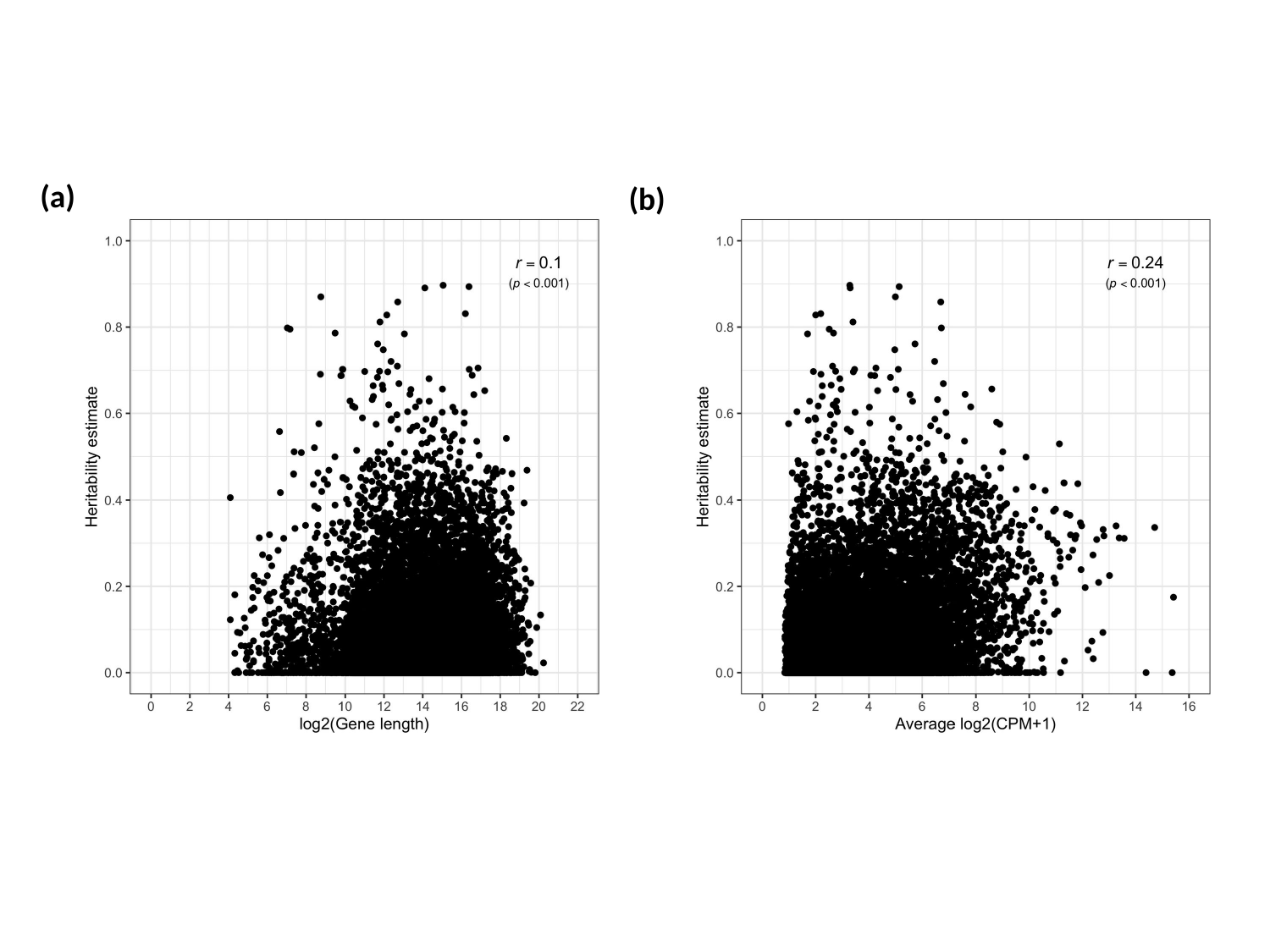

(a)
(b)

Supplement: Supplementary file 1 — Additional file 1: Figure S1. Relationship of the estimates of heritability of gene expression with gene length (a) and average expression level (b). [file 12711_2023_860_MOESM1_ESM.pptx]

## Slide 1
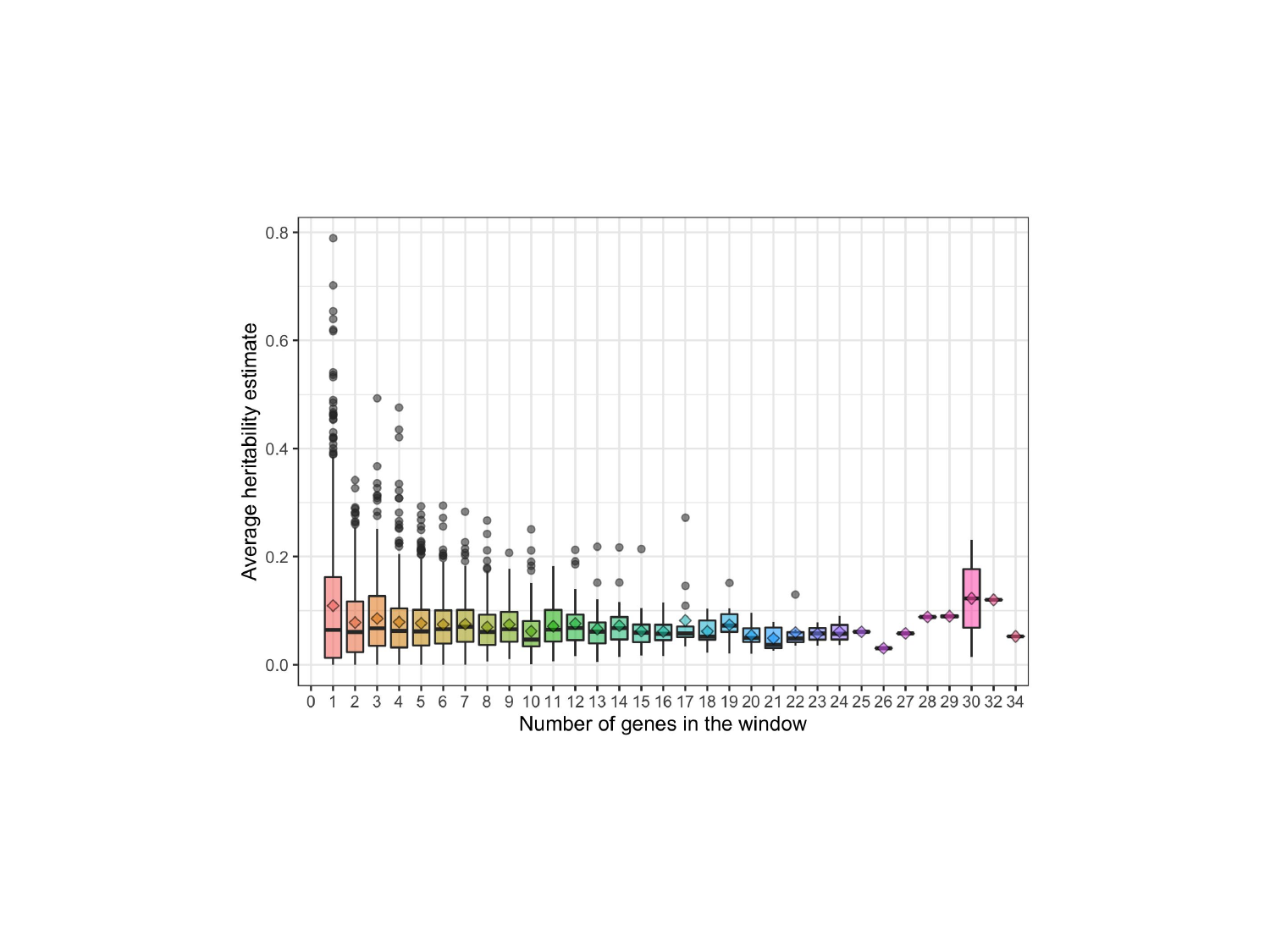

Supplement: Supplementary file 2 — Additional file 2: Figure S2. Box blots of average heritability estimates of genes in 0.5-Mb non-overlapping windows across the genome, versus the numbers of expressed genes within the window. [file 12711_2023_860_MOESM2_ESM.pptx]

## Slide 1
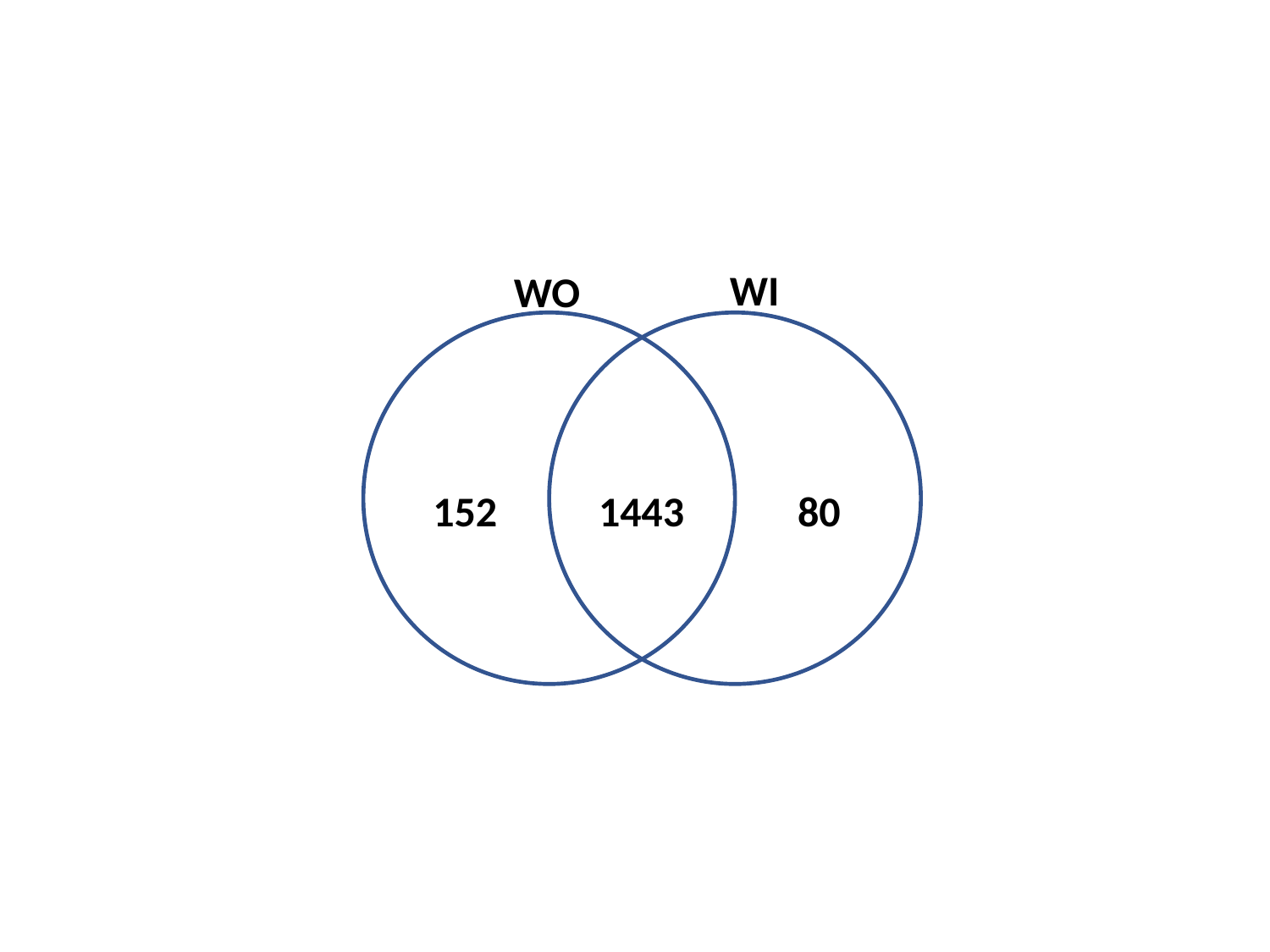

WI
WO
1443
152
80

Supplement: Supplementary file 3 — Additional file 3: Figure S3. Number of genes (n = 1675) with expression levels that had estimates of heritability of 0.2 or higher in the models with (WI) or without (WO) accounting for the cell composition. [file 12711_2023_860_MOESM3_ESM.pptx]
